# Microfiltration Membrane Pore Functionalization with Primary and Quaternary Amines for PFAS Remediation: Capture, Regeneration, and Reuse

**DOI:** 10.3390/molecules29174229

**Published:** 2024-09-06

**Authors:** Sam Thompson, Angela M. Gutierrez, Jennifer Bukowski, Dibakar Bhattacharyya

**Affiliations:** 1 Department of Chemical and Materials Engineering, University of Kentucky, Lexington, KY 40506, USA; sam.thompson@uky.edu (S.T.); jennifer.bukowski@uky.edu (J.B.); 2 Sustainability and Analytical Equipment Facility, University of Kentucky, Lexington, KY 40506, USA; amgu232@uky.edu

**Keywords:** in situ functionalization, polyvinylidene difluoride, polyether sulfone, quaternary and primary amines, PFAS sorption, responsive membrane

## Abstract

The widespread production and use of multi-fluorinated carbon-based substances for a variety of purposes has contributed to the contamination of the global water supply in recent decades. Conventional wastewater treatment can reduce contaminants to acceptable levels, but the concentrated retentate stream is still a burden to the environment. A selective anion-exchange membrane capable of capture and controlled release could further concentrate necessary contaminants, making their eventual degradation or long-term storage easier. To this end, commercial microfiltration membranes were modified using pore functionalization to incorporate an anion-exchange moiety within the membrane matrix. This functionalization was performed with primary and quaternary amine-containing polymer networks ranging from weak to strong basic residues. Membrane loading ranged from 0.22 to 0.85 mmol/g membrane and 0.97 to 3.4 mmol/g membrane for quaternary and primary functionalization, respectively. Modified membranes exhibited a range of water permeances within approximately 45–131 LMH/bar. The removal of PFASs from aqueous streams was analyzed for both “long-chain” and “short-chain” analytes, perfluorooctanoic acid and perfluorobutyric acid, respectively. Synthesized membranes demonstrated as high as 90% rejection of perfluorooctanoic acid and 50–80% rejection of perfluorobutyric acid after 30% permeate recovery. Regenerated membranes maintained the capture performance for three cycles of continuous operation. The efficiency of capture and reuse can be improved through the consideration of charge density, water flux, and influent contaminant concentration. This process is not limited by the substrate and, thus, is able to be implemented on other platforms. This research advances a versatile membrane platform for environmentally relevant applications that seek to help increase the global availability of safe drinking water.

## 1. Introduction

Human and industrial activities contribute to the contamination of the global water supply in part through the release of anthropogenic chemicals into wastewater and process runoff. As our understanding of the widespread negative effects of such contaminants grows, so does our commitment to developing remediation techniques to properly cleanse water [1,2,3]. One of the currently prominent classes of widespread and resilient wastewater contaminants is halogenated carbon compounds, specifically fluorinated alkyl chains.

Per- and polyfluorinated alkyl substances (PFASs) are a family of manmade congeners with dual hydro- and oleo-phobic properties that are developed and utilized for their advantageous properties for manufacturing water-, temperature-, and friction-resistant materials [4]. PFASs have been widely used in consumer products and industrial processes for decades as cleaning agents, degreasers, and firefighting foams, among others [5]. Unfortunately, such compounds are known to resist degradation by natural means and are easily transported through environmental cycling processes, resulting in their environmental persistence, which has been a cause of concern for potential toxic human health effects that arise from their bioaccumulation and has presented a grave concern for remediation efforts [6,7,8,9]. These properties are largely attributed to the strong and stable carbon–fluoride bond, whose bond strength of 440.99 kJ/mol resists degradation in ambient conditions [10]. In addition to the prevalence and difficulties of “legacy” PFASs, such as the longer eight-carbon chain perfluorooctanoic acid (PFOA) and perfluorooctane sulfonic acid (PFOS), the environmental community continues to recognize novel and interrelated fluorinated compounds, including short-chain alkyls and ester-linked varieties. An emerging understanding of these contaminants drives the need for fundamental research into their removal mechanisms from water to maintain clean drinking water globally.

Although all PFASs are composed of fluorinated alkane carbon chains and some potentially charged terminal group, their physicochemical properties can vary dramatically depending on the specific chemical composition of each species [11]. The differences in water solubility, pK_a_, partition coefficient, etc., are largely due to the length of the hydrophobic fluorinated chain or identity of the terminal end group [12]. This leads to variable performances in contaminant remediation [13,14]. For example, PFAS rejection by nanofiltration or reverse osmosis membrane systems ranges around 90–99% for long-chain analogs but widens to 50–99% for short-chain compounds [15].

Conventional water treatment technology comprises separation and destruction techniques. Current treatment technologies used at water and wastewater treatment plants (e.g., coagulation, flocculation, aeration, sand/rapid filtration, sedimentation, and disinfection) are inefficient at removing and incapable of destroying PFASs from contaminated groundwater and wastewater [16,17]. Furthermore, recent EPA regulations have established a challenging 4 ppt maximum contaminant level, which demands robust separation and detoxification efforts [18]. Developing remediation strategies are rapidly being explored for their applicability. A list of example environmental PFAS levels and potential filtration or degradation technologies are shown in Table 1. Total removal or destruction relies on remediation techniques that can be energy intensive with high operational costs or generate toxic byproducts from the parent contaminants [19].

Thus, efforts to concentrate residual streams could alleviate remediation bottlenecks by reducing the overall energy or operational cost of separation or by allowing cleaner degradation methods to be employed due to a more manageable residual volume. Notably, the literature lacks efforts that result in an elevated concentration of PFASs in the residual stream.

Despite known limitations, ion-exchange or adsorptive resins have become a mainstay in PFAS remediation efforts. The adsorption of PFASs has been widely incorporated and studied, resulting in a well-characterized sorption behavior and a mechanistic understanding of PFAS interactions [32,33]. Such an analysis has elucidated that electrostatic interaction is one of the major attractive forces for PFAS adsorption, suggesting that ion-exchange resin or polymer adsorbents may achieve higher removal capacities [14,34,35]. However, typical anion-exchange systems have mass transfer limitations due to the nature of diffusion to the active surface of the resins [36]. The use of membranes has been posited to alleviate many of these concerns.

Membranes as separation systems are capable of size exclusivity by virtue of their porous structure, and these pores can be further functionalized to act as a channel of active sites through which water is passed [37,38,39]. When the pores are functionalized, convective flow bolsters the mass transfer kinetics of potential contaminants to boost the overall performance of the separation process. Localized polymerization within the pore network of the membrane is capable of this combined performance benefit. Polymeric membranes functionalized in this way can also introduce stimuli-responsive properties [37].

The incorporation of amine groups provides a method that includes effective removal and regeneration potential with permeability benefits of membranes, as evidenced by small-scale tests capturing influent PFAS concentrations of 30–300 ng/L (0.03–0.3 ppb) [29]. Wholistic analysis of removal techniques has suggested that for a goal of 25 ng/L discharge, membrane adsorption would cost around 0.87 USD/m^3^, compared to regular ion-exchange adsorption costing between 1.2 and 8.9 USD/m^3^ [40]. Many commercially available ion-exchange resins on the market utilize some form of amine [14,41,42]. Depending on the number of carbons bonded to the nitrogen atom, the resulting polymer has a range of isoelectric points that can be leveraged when capturing and regenerating, and furthermore, these small changes may also influence the resulting affinity for various types of PFASs [43]. Our recent work has demonstrated the adsorption and desorption of PFOA from simulated groundwater using quaternary and tertiary amines grafted to polymer membranes, accomplishing over 80% removal even after multiple cycles [44]. Secondary and primary amines are known to be similarly applicable, but their performances in polymeric membranes for PFAS remediation have not been fully elucidated [45].

**Figure 1:**
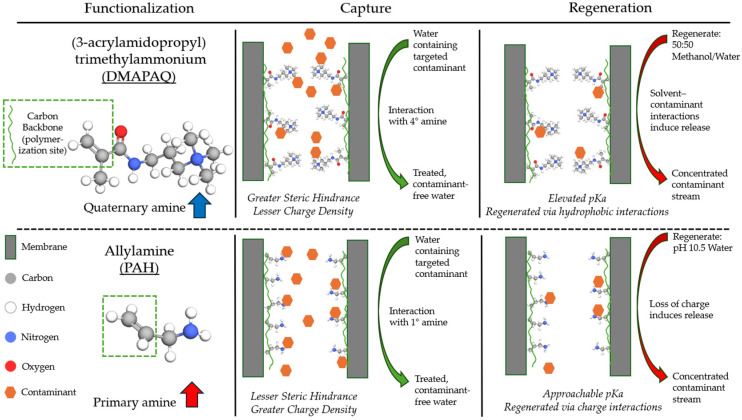
Schematic of functionalization monomers used for membrane synthesis, targeted contaminant capture, and subsequent regeneration. Not to scale.

This work aims to test and analyze the application of two types of anion-exchange functionality within a membrane platform to adsorb (via site interactions) and concentrate emerging contaminants of concern (i.e., PFASs). A schematic overviewing the synthesis, capture performance, and regeneration mechanism is shown in Figure 1. While our group and others have studied similar mechanisms, the use of primary and quaternary amine-containing anion-exchange resins in a functionalized microfiltration membrane platform has not been well explored in the field of PFAS remediation. Specifically, research was conducted to address three goals: 1. develop and optimize a synthesis procedure that utilizes aqueous chemistry to introduce ion-exchange groups that are known to be effective for the sorption of certain PFAS species into a membrane; 2. analyze the separation performances of primary and quaternary amine-based membranes with short- and long-chain perfluorinated carboxylic acids; and 3. analyze the regeneration potential of functionalized membranes for both primary and quaternary amine groups.

## 2. Results and Discussion

Pore-functionalized membranes were successfully prepared utilizing green and scalable chemistry processes to introduce cationic amines into membrane pores for enhanced PFAS removal performance. Polyvinylidene difluoride (PVDF) and polyether sulfone (PES) membranes served as the platform for the in situ polymerization of the primary and quaternary amines containing chemicals allylamine hydrochloride (PAH when polymerized) and (3-acrylamidopropyl) trimethylammonium (DMAPA-Q). Throughout this work, the quaternary amine-containing DMAPA-Q will be represented in blue, while the primary amine-containing PAH will be represented in red. Successful polymerization and successful functionalization were validated through the measurement of chemical bonds using Fourier-transform infrared (FTIR) spectroscopy, pure water permeance by measuring the flow rate over time, and contaminant capture using liquid chromatography paired with mass spectrometry (LC/MS/MS). The regeneration capacity of the developed membranes was evaluated using specific regeneration media for each membrane, and the reusability potential for their repeated use was measured for three cycles.

### 2.1. Pore Functionalization

Commercial microfiltration membranes were functionalized through the in situ free radical polymerization of quaternary or primary amine-containing monomers with a crosslinker, a process well described in the previous literature [37,39,46,47]. This was achieved by using vacuum filtration to coat the membrane pores with a solution containing the desired monomer, a crosslinker, and an initiator. Excess solution was then removed from the membrane surface, and the membrane was heated at 85 °C in a vacuum oven to initiate the polymerization by cleaving a single bond in the initiator to form free radicals. To prevent the deformation of the membranes in the oven, they were placed between two thin plastic sheets and clamped together between a Teflon plate and glass plate. Once functionalized, the membrane surface was rinsed with deionized water and dried overnight prior to use. This process is illustrated in Supplementary Figure S1. The introduction of amine-containing functional groups via the polymeric network formed in the membrane pore creates a matrix of anion-exchange material capable of ion exchange with the passing contaminants dissolved in the water permeate. In contrast to membranes containing quaternary amine residues, primary amine anion exchangers can more readily change charge at higher pH levels. This response to changes in the operation parameters potentially allows for the robust regeneration of functionalized membranes at feasible industrial conditions by avoiding the use of harsh chemicals or electric fields [48,49]. The loading of quaternary and primary amine polymeric materials is described in Table S1. The presence and identification of the polymer can be verified by testing for the chemical bonds encountered on the surface of the membrane.

**Figure 2:**
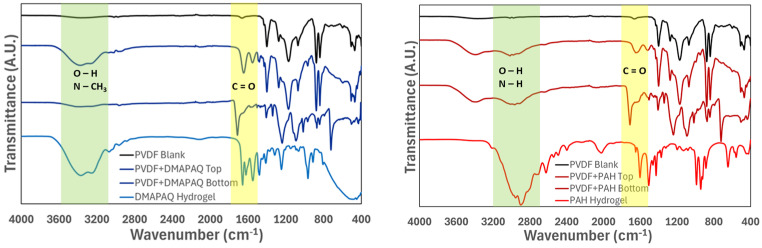
Graph of transmittance intensity attained using Fourier-transform infrared spectroscopy of isolated polymer hydrogel and functionalized membranes. Coordination of transmittance peaks corresponds to presence of chemical bonds.

The absorbance or transmittance of infrared light when passed through a sample can be measured to evaluate the presence of certain chemical bonds using FTIR spectroscopy. The membranes functionalized with quaternary and primary amine-containing polymers were analyzed to verify the successful incorporation of functionalized hydrogel into the membrane. PVDF membrane spectra have peaks in the “fingerprint” region (500 to 1500 cm^−1^) that are indicative of C-H and C-F bonds. After in situ functionalization of the membrane pores, the evaluated membranes showed peaks around 1700 cm^−1^ and between 3300 and 3400 cm^−1^, which indicate C=O double bonds and O–H or N–H bonds, respectively. The FTIR spectra for the quaternary and primary amine-functionalized PVDF membranes, the bulk polymer, and a pristine PVDF membrane sample are shown in Figure 2.

The characterization of the synthesized membrane enabled the validation of cationic polymer functionalization and pore maintenance. FTIR spectrum analysis, shown in Figure 2, corroborates the polymerization of each polymer in the commercial membrane. In each graph, the pristine PVDF membrane exhibits only the presence of C–H and C–F bonds. In the functionalized membranes, transmittance peaks are seen as being in agreement with the peaks found in the FTIR spectra of both polymers (e.g., O-H stretching (3550–3200 cm^−1^), C=O (1600 cm^−1^), amine salt (3000–2800 cm^−1^), N-H bending (1650–1580 cm^−1^), and C-N stretching (1250–1020 cm^−1^)). The FTIR spectra of the polymer synthesized under identical conditions is shown on the bottom of each figure. This signature highlights the difference between the chemical bonds present in the quaternary and primary amine-containing polymers. The intensity of the broad transmittance peak around 3000 cm^−1^ is likely due to the presence of water in the hydrogel-forming polymer.

### 2.2. Surface Characterization

Before making conclusions about the adsorptive performance or ion-exchange capacity, it is important to ensure the functionalization process is not destructive to the membrane structure. Scanning electron microscopy (SEM) enabled the validation of the pore integrity and overall membrane structure. Microfiltration membranes can have symmetric or asymmetric pores depending on their composition and method of synthesis. This porosity extends throughout the thickness of the membrane. Compared to other commercially relevant membranes, microfiltration membranes have large pore sizes that often exist in the 100 nm range but can be as large as 1 micron.

**Figure 3:**
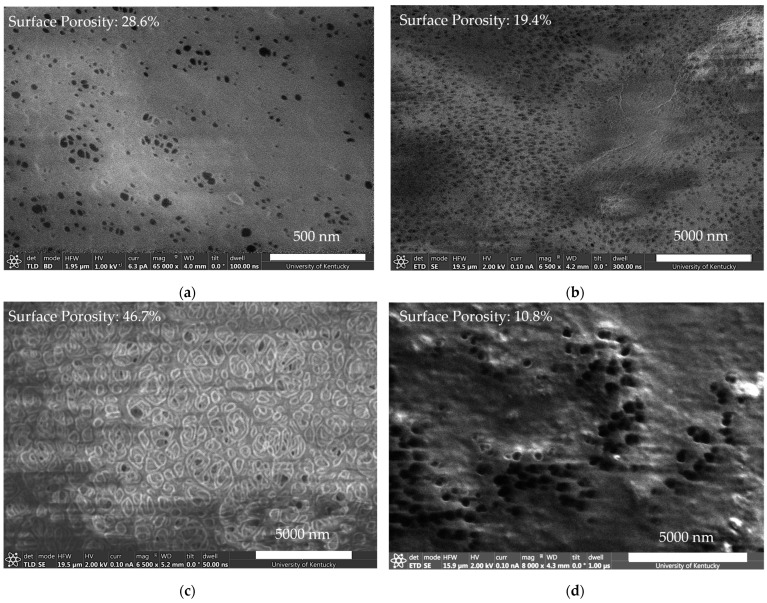
Scanning electron microscopy of functionalized membrane surface. (**a**) Pristine PVDF membrane, scale bar 500 nm; (**b**) larger view of PVDF membrane functionalized with DMAPA-Q, scale bar 5 µm; (**c**) hydrophilized PES membrane functionalized with DMAPA-Q, scale bar 5 µm; (**d**) PVDF membrane functionalized with PAH, scale bar 5 µm.

Lighter regions are due to charge buildup on the electrically resistant polymeric surface. Dark voids are indicative of pores viewable from the surface. The apparent average pore size in the PVDF membrane around 50 nm is consistent with previous observations of 62.2 nm, with the largest pores reaching 300–400 nm [38]. PES membranes exhibited much larger, asymmetric pores that agree with manufacturer specifications of a 450 nm average pore diameter. This shows that the functionalization performed is possible despite variations in the pore size of the membrane substrate. Although polymer incorporation made imaging more difficult, similar pore sizes were seen before and after functionalization. Cross-sectional images on similarly functionalized PVDF650 membranes can be seen in prior work [47]. Figure 3 shows that the surface porosity was maintained despite the addition of DMAPA-Q into the pore network of the membrane. Alternatively, microfiltration membranes functionalized with primary amine exhibited some coverage of the surface pore network, resulting in new, larger surface pores. This functionalization also has an impact on the surface charge. Previous work by our group has shown the inclusion of amine-containing polymers to a functionalized membrane results in a positive zeta potential on the surface [50]. Additionally, this functionalization alters the hydrophilicity of the membrane. PVDF650 functionalized with primary and quaternary amines exhibited water contact angles of 22° and 58°, respectively. The contact angle’s decay over time, as shown in Figure S2, suggests that the primary amine has a significantly higher wettability. PVDF membranes were chosen for their chemical resilience and industrial competence, albeit not required for the remediation platform. PES membranes were similarly functionalized to demonstrate the versatility of the functionalization process.

### 2.3. Membrane Performance

After the primary and quaternary amine functionalization was performed, the water flux was measured for three separate pressure differentials to analyze the water permeance. Modified commercial membranes exhibited a notable decrease in the water flux. The permeance is often cited in liters of water per square meter of membrane per hour (LMH) passing through the membrane per unit of bar pressure; therefore, the linear regression of the reported water flux data provides a characterization of membrane water permeability. A graph of functionalized membranes and their pure water permeance can be seen in Supplementary Figure S3. DMAPA-Q-functionalized PVDF400 membranes exhibited around 51 LMH/bar, a 75% reduction in permeability when compared to the stock commercial PVDF400 membrane. Following the functionalization procedure, a reduction in water flux and the introduction of amine groups both indicated the successful incorporation of the polymer network within membrane pores. Water permeance is a key metric in the performance of microfiltration and ultrafiltration membranes. A reduction in the overall water flux after functionalization indicates successful polymerization within the membrane pore network. PVDF membranes, when pristine, offer very high water permeance. PVDF650 differs from PVDF400 by having a larger average pore diameter. Thus, you can expect PVDF650 to have a relatively higher flux overall.

Another critical factor for the viability of this membrane is its operability across a range of pH levels. Again, the pure water flux of the membrane was analyzed at various feed acidity levels to understand the effects that the polymer may exhibit in their response.

**Figure 4:**
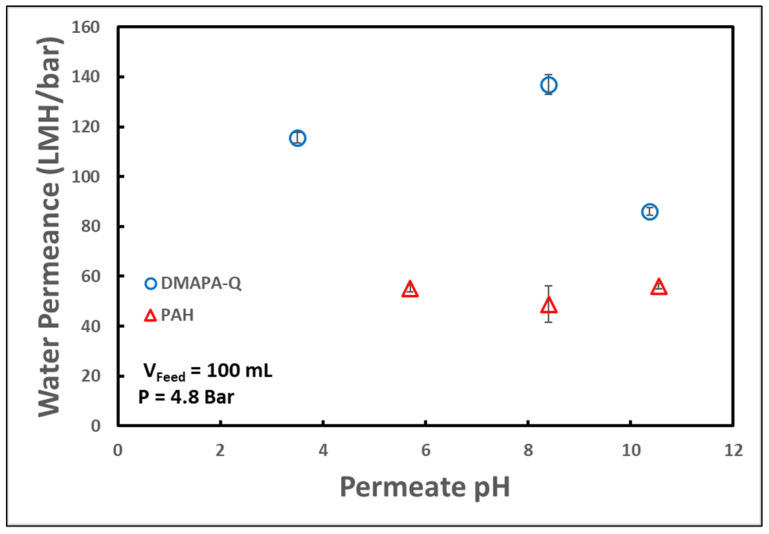
Effect of feed pH on water permeance. Permeation operated in dead-end batch conditions. pH adjusted with hydrochloric acid or sodium hydroxide. Error bars represent +/− one standard deviation in series of permeance measurements, *n* = 3.

As seen in Figure 4, the PAH-functionalized membrane had a lower water permeance than the DMAPA-Q-functionalized membrane at all pH ranges. Notably, the quaternary and primary amine polymers maintained pure water permeance across the range of pH levels studied. Of course, it should be noted that water permeability will be a strong function of the extent of functionalization and the cross-linking density. A common concern with more stringent membrane separations, such as ultrafiltration or nanofiltration, is membrane surface fouling, or the obstruction of pores by filtering solutes. In order to understand how functionalized membranes would perform in the presence of foulants, membrane water permeance was measured before and after the introduction of 0.5 mg/mL negatively charged dye (orange II dye, MW 350). Compared to a 2.8% reduction in membrane flux in pristine PVDF650 membranes, quaternary and primary amine-functionalized membranes showed 1.2% and 46% reductions, respectively. This suggests that membranes functionalized with DMAPA-Q had insignificant impact on membrane fouling. On the other hand, allylamine functionalization showed a significant reduction in flux, although likely not due to fouling primarily. As primary functionalization resulted in a greater extent of amine loading, additional capture contributes to less water permeance via the occupation and obstruction of active sites in the polymer matrix. This change in hydrogel morphology decreases the water corridor in the membrane pores. Primary amine functionality brings in another versatility in terms of conformation change through pH, which could potentially lower surface fouling.

**Figure 5:**
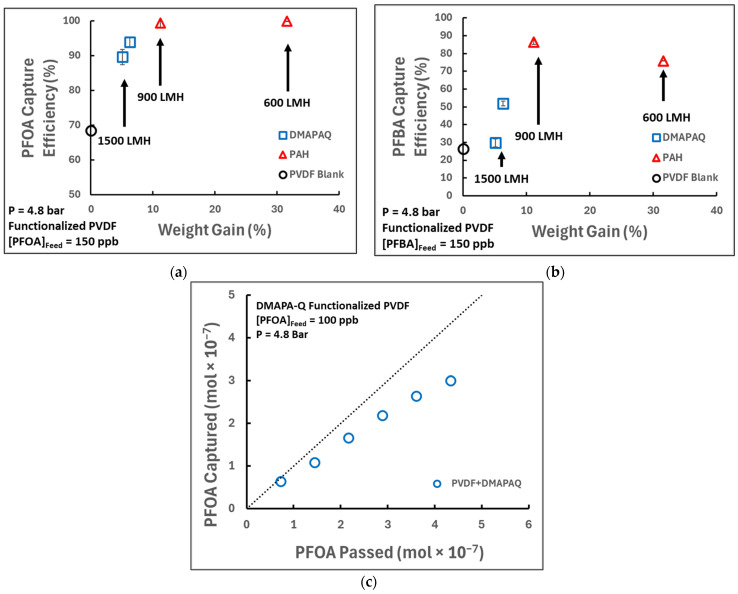
PFOA/PFBA capture by primary and quaternary amine-functionalized PVDF650 membrane and pristine PVDF650 blank membrane using dead-end batch filtration. Capture efficiency is defined as ((feed concentration—permeate concentration)/feed concentration) × 100%. Permeate represents 30% water recovery. (**a**) PFOA capture efficiency; (**b**) PFBA capture efficiency; (**c**) PFOA capture efficiency during processing; dotted 45° line represents capture efficiency of 100%. Error bars represent +/− one standard deviation using technical triplicates; for PVDF blank capture, *n* = 1.

Important to this study is the performance of the anion-exchange polymer when different species of PFASs are permeated through the membranes. We hypothesized that due to differences in the hydrophilicity and steric hindrances, short-chain analogs will be captured at a lesser rate to that of their longer-chain counterparts. Initially, the functionalized membranes were tested for their ability to capture PFOA and PFBA at a 150 ppb (150,000 ng/L) concentration under approximately 5 bar transmembrane pressure, which was selected to be representative of the contaminant levels seen in residuals from remediation efforts and potential industrial operating conditions. The permeate was then measured for levels of PFOA and PFBA using LC/MS/MS, as shown in Figure 5.

In general, the capture of PFASs improved with additional loading of the membrane. This is expected, as a greater weight gain corresponds to a greater number of amine sites present. Additionally, higher water flux through a membrane creates less residence time, so the increased loading may further enhance capture by allowing the contaminants more time to interact with the membrane. Initial capture seems to follow near 100% removal of PFOA but falls off over time, showing that as sites are being used to capture PFAS molecules, less sites are available.

A common concern for altered membrane systems is additive leaching, or the release of chemicals from the membrane matrix into the permeate stream. The feed and permeate for each membrane were studied for the potential presence of contamination. As expected, it seems that any residual component of either unpolymerized reagent or unsecured polymer resin is removed by rinsing immediately after synthesis. This is evidenced by initial permeate streams resembling the spectrum of the unaltered feed stream, which can be seen in Figure S4. This has some implication on the long-term stability of the functionalized membrane, as polymerized anion-exchange material is withheld by the membrane porosity. Furthermore, the resemblance of the synthesis scheme and polymerization reaction to our previous work suggests the chemical alterations should be maintained throughout the lifespan of the membrane, as evidenced by the detection of characteristic N–H, C=O, and C–F stretching using attenuated total reflectance–FTIR after extended (8–12 h) operation and multiple (4–9) regeneration cycles [51]. Importantly, the functionalized membranes studied in our current work maintained water permeance and certain capture performance characteristics throughout multiple cycles.

### 2.4. Regenerative Aspects

The regeneration of remediation technology is a tremendous hurdle to the long-term implementation of effective solutions [29,32,33,52]. Moreover, conventional technologies are ineffective at targeted concentrations during the regeneration process, relying on incineration or landfilling to treat the process residuals [2,40,53,54]. By leveraging the separation potential of sub-micron membrane filtration and selectivity of pH-responsive ion exchange, these anion-exchange membranes provide a robust platform for capturing and concentrating desired compounds, in this case PFASs.

**Figure 6:**
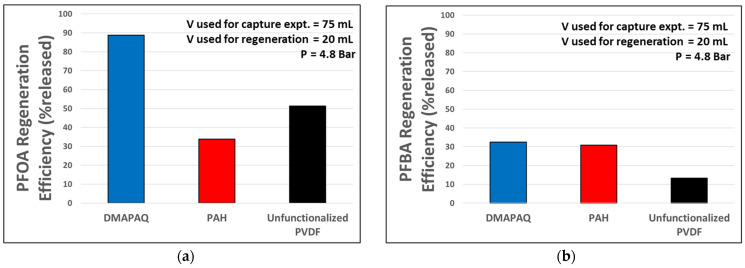
Regeneration efficiency of functionalized anion-exchange membranes, defined as percentage of captured PFASs released in approximately 25% regeneration volume. DMAPAQ-functionalized PVDF and unfunctionalized PVDF regeneration stream employed a 50:50 methanol and water mixture, while PAH-functionalized PVDF regeneration stream used water with a pH of 10.5. (**a**) PFOA concentration in permeate using regeneration stream; capture experiment employed 120 ppb PFOA; (**b**) PFBA concentration in permeate using regeneration stream; capture experiment employed 140 ppb PFBA. All experiments were performed at 4.8 bar (LMH/bar for functionalized membrane = 50–120); V in captions indicates volume, *n* = 1. PFOA analytical error: +/−5.5%; PFBA analytical error: +/−6.5%.

Each membrane was tested for its ability to release captured PFASs using a regenerative feed solution. For the quaternary amine and pristine PVDF membranes, a 50:50 mixture of methanol and water was passed through the membrane at 5 bar. In the case of the primary amine membrane, the regeneration feed consisted of water elevated to a pH of 10.5 by the addition of NaOH. Figure 6 depicts the ability for each tested membrane to perform the controlled release of captured contaminants.

The regeneration efficiency is reported as the percentage of PFOA or PFBA released compared to the amount theoretically captured. The PFOA and PFBA concentrations were determined using LC/MS/MS. Ion-exchange materials are most often regenerated using a high-concentration solution containing sodium chloride or sodium hydroxide, with the intent of displacing the captured contaminants and reverting resins or polymers into their chloride or hydroxide form. Other regeneration techniques include passing a water and methanol mixture or weak acid, but these have the downside of potentially damaging the membrane or polymer. Some studies have proposed alternative feeds, such as acetone, with demonstrated benefits in the regeneration of sorbents that captured PFASs containing carboxylic acid groups compared to when PFASs containing sulfonic acid was similarly adsorbed, although its lower vapor pressure could contribute to the unwanted aerosolization of smaller carbon-chain PFASs [55,56]. By incorporating a primary amine, the functional group pKa value can be leveraged to encourage the desorption of anionic contaminants by using a regeneration stream with an elevated pH. The quaternary amine-functionalized membrane maintains its positive charge across a much larger pH range, so a 50:50 methanol-to-water mixture was used for regeneration.

The regeneration efficiency reflects the ability for each membrane to release the captured contaminant. When PFOA was passed through each membrane, the functionalized membranes were able to capture more than the unfunctionalized PVDF membrane. Based on experimental data with a 120 ppb (120,000 ng/L) feed and 75 mol water permeation through 14.6 cm^2^ membrane, the DMAPAQ and PAH were able to capture 7.4 µg and 9 µg of PFOA compared to the pristine PVDF capture of 5 µg. When regenerated, the DMAPAQ, PAH, and pristine membranes produced concentrates of PFOA at approximately 328 ppb, 152 ppb, and 128 ppb, respectively. While the PAH-functionalized membranes exhibited the highest rates of capture, the regenerating feed solution seems to not be sufficient for optimum release. For PFBA, both quaternary and primary amine-functionalized membranes exhibited regeneration efficiencies greater than pristine PVDF microfiltration membranes, resulting in regeneration stream concentrations of 57.5 ppb, 138.5 ppb, and 8.5 ppb, respectively. Of course, these concentrations depend on the amount captured. Here, it seems the shorter-chain PFBA is less amenable to methanol and water recovery. The PFOA results could be indicative of how the lengthened hydrophobic carbon chain plays an increased role in interactions with the membrane, as opposed to the lesser carbon chain of PFBA, where its charge could see a larger relative impact.

### 2.5. Reusability

In order to show the viability of membrane application in industrially demanding sectors, the membranes underwent three cycles of PFAS treatment, regeneration, and reuse. Each membrane was primed with 23,500 mg/L NaCl before being used to treat water containing around 100 ppb (100,000 ng/L) of both PFOA and PFBA.

In each cycle, the membrane was challenged with a mixture of 120 ppb PFOA and 140 ppb PFBA, and subsequently, the feed was changed to the regeneration stream. The permeate and regeneration streams were collected and analyzed using LC-MS/MS. Increasing concentrations of permeate streams in the green regions exhibit the interaction between the contaminant and the membrane. On the other hand, in the regeneration stream, desorbed PFOA or PFBA can be seen entering the PFAS-free feed. An effective membrane maintains its capture efficiency over time and is resilient over multiple cycles. Each membrane was used to filter a PFAS solution containing approximately 120 ppb PFOA and 140 ppb PFBA. A mixture was used to better understand how differences in the contaminant resulted in different capture performances within the same membrane. Both PAH- and DMAPA-Q-functionalized membranes demonstrate the successful removal of PFOA from the feed water stream, with around 99% and 90% capture efficiencies, respectively. As expected from the literature, the performance dropped for the short-chain PFBA. The regeneration results followed similar trends, except in the pristine membrane experiment. The pristine membrane was able to release a higher concentration of PFOA in the regeneration stream when compared to the PAH-PVDF membrane, while the reverse was true for PFBA. This highlights the impact of molecular hydrophobicity on the capture and regeneration performances, as the increased interaction and regeneration is likely due to hydrophobic regions of the pristine PVDF membrane.

**Figure 7:**
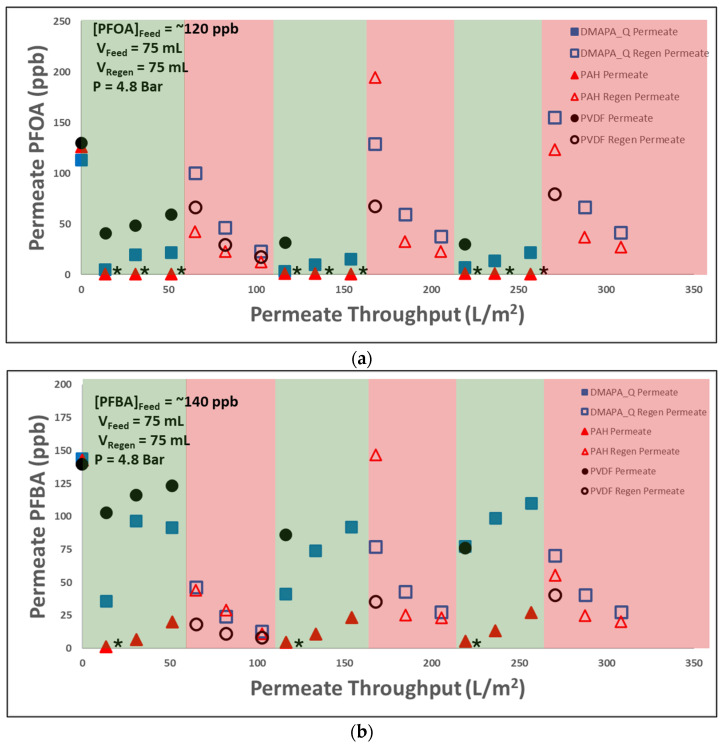
Capture and controlled release of (**a**) 120 ppb PFOA solution and (**b**) 140 ppb PFBA solution in functionalized and pristine membranes. Filled-in symbols indicate concentration of PFASs detected in permeate. Open symbols indicate concentration of PFAS detected in the regeneration stream. Green region indicates permeate from ~130 ppb PFOA/PFBA mixture, red region indicates permeate from regeneration stream. DMAPA-Q and PAH membranes were regenerated with 50:50 methanol/water mix and water adjusted to pH 10.5, respectively. All permeations were performed at 4.8 bar under neutral conditions, *n* = 1. * Denotes measurement below instrumentation limit of quantification.

The repeated trends shown in Figure 7 provide a pattern into the mechanistic adsorption and desorption of the contaminant. Initial high capture is slowed down as contaminants reach and electrostatically interact with the active sites in the membrane. Notably, the capture performance in both membranes recovered to near-initial levels after regeneration. This suggests the capture and release is not negatively affecting the functionalized pore matrix. The ability of remediation membranes to maintain their capture performance at concentrations as low as 100 ppb (100,000 ng/L) is vital for application in the PFAS remediation field. Also, regeneration seemed to occur rapidly and, in some cases, resulted in PFAS concentrations greater than the initial processed feed. This indicates the potential for rapid controlled release of captured PFASs. The ability to concentrate residual contaminant streams serves to reinforce conventional separation technologies as well as offers a solution to many concerns of promising degradation technologies.

This study makes progress towards better understanding PFAS remediation alternatives through the synthesis and application of pore-functionalized microfiltration membranes. Commercial membranes can be functionalized with varying amounts of primary or quaternary amine-containing anion-exchange polymers for the capture of two newly regulated PFAS compounds: PFOA and PFBA. Despite promising results, the performance characteristics of such membranes could be better analyzed through modifications of the processed water matrix. Our previous work has shown that conditions such as ionic strength, ion properties, and the presence of natural organic matters can negatively impact the capture of PFOA, including a decrease in the PFOA removal percentage from 96% to 85% in the presence of 20 mM NaCl and from 96% to 72% in the presence of 10 mg/L organic matter (fulvic acid) [44]. To evaluate the impact of co-contaminants on functionalized PVDF microfiltration membranes, PFOA capture was also analyzed when the feed was supplemented with two additional conditions: the inclusion of 20 mM NaCl and the inclusion of 10 mg/L humic acid. The membranes tested had a similar extent of amine loading, ranging from 0.39 to 0.43 mmol amine/g membrane. Interestingly, the apparent capture efficiency by quaternary amine-loaded membranes of around 90% PFOA removal, as seen in Figure 5, was reduced to 53% in the presence of 20 mM NaCl and boosted to 98% in the presence of humic acid. Because of the stark difference in capture with other ions present, the competing anions do seem to negatively impact the ability to capture long-chain PFASs. The addition of humic acid could have given the impression of better separation, when in actuality, the PFOA may be interacting with the organic matter once in the polymer matrix. The introduction of additional PFASs or other contaminants alongside common wastewater components, such as organic matter, dissolved solids, or ions, could all contribute to evaluating the feasibility in industrial applications. Extending PFAS processing experiments through larger operating volumes or continuous tangential flow filtration would also provide better insight into the long-term viability and potential PFAS breakthroughs. Additionally, other methods of regeneration could elucidate optimized conditions for reuse.

## 3. Materials and Methods

### 3.1. Materials

The following chemicals were used for the synthesis and characterization of the functionalized membranes: ASTM Type 1–Type 2 water (ACS reagent grade, RICCA), ammonium persulfate, N,N′-Methylenebisacrlyamide (99+%, ThermoScientific, Waltham, MA, USA), (3-acrylamidopropyl) trimethylammonium chloride stabilized (DMAPA-Q) (74%–76% in water, TCI, Portland, OR, USA), allylamine chloride (TCI, Portland, OR, USA), sodium perfluorooctanoic acid, and heptafluorobutyric acid. PV650 and PV400 polyvinylidene difluoride membranes (178 µM thickness) were provided by Solecta Inc. (Oceanside, CA, USA) PES membranes refer to Millipore Express PLUS membrane filter polyether sulfone with 0.45 µM pore-size membranes (165 µM thickness).

### 3.2. In Situ Pore Functionalization

The synthesis of an ion-exchange polymer inside the membrane pores was performed using a simple functionalization approach. Briefly, a solution containing monomers was passed through microfiltration membranes using vacuum filtration and then polymerized via temperature-initiated free radical polymerization. Leftover monomer solution was collected in scintillation vials and similarly polymerized to verify gelation. Following the application of 85 °C heat in a vacuum oven for 2 h, the membranes were rinsed with deionized water and dried.

### 3.3. Characterization of Surface Morphology

Surface imaging and cross-sectional analysis were conducted using a focused ion beam/scanning electron microscope (FIB/SEM) dual beam (Helios Nanolab 660, FEI/ThermoFisher Scientific, Waltham, MA, USA). Surface porosity analysis was performed using ImageJ particle analysis calculations.

Contact angle measurements were performed using 10 µL of deionized water with the Krüss DSA100S drop shape analyzer (Matthews, NC, USA).

The analysis of the elemental composition was performed through Fourier–transform infrared spectroscopy (Nicolet iS50 FT-IR, ThermoScientific, Waltham, MA, USA). Wavelength transmittance was measured with averages of 32 scans using a potassium bromide crystal detector.

### 3.4. Characterization of Membrane Performance

Permeability studies were performed using stainless steel Sterlitech (Auburn, WA, USA) P/N 4750 dead-end filtration cells with a membrane active area of 14.6 cm^2^. The polymers were initially made to perform ion exchange in their chloride form using 23,500 mg/L NaCl solution. For permeance data at varying pH levels, the pH was adjusted using hydrochloric acid or sodium hydroxide to reach the desired acidity or alkalinity, respectively.

Anti-fouling studies were performed using glass normal flow filtration (NFF) stirred cell units from Millipore Sigma (Burlington, MA, USA). Membranes were cut to 40 cm^2^ circles and used to filter deionized water or deionized water containing 0.5 mg/mL orange II dye from Acros Organics (Geel, Belgium).

The permeate was collected in 10 mL intervals for subsequent analysis. Performance with perfluorinated compounds was analyzed through PFOA and PFBA permeation studies. For PFAS filtration tests, the concentrations were in the 1–200 ppb range. Co-contaminant studies were performed with similar conditions with the addition of 10 mg/L humic acid or 20 mM NaCl to a PFOA solution. The regeneration of ion-exchange moieties was performed by passing a 50:50 methanol and water mixture or water with the pH adjusted to 10.5 for quaternary amine and primary amine resins, respectively.

### 3.5. Perfluorinated Compound Analysis

PFAS analysis was performed using liquid chromatography–triple quadruple mass spectroscopy (LC/MS/MS Agilent 6470 LC/TQ, Santa Clara, CA, USA) with a PFC-free kit. Chromatography used LCMS-grade water with 10 mMol ammonium acetate and LCMS-grade methanol as its aqueous and organic phases, respectively. For mass spectroscopy of PFOA and PFBA, EPA method 533 was followed, with some exceptions. The sheath gas temperature was set to 350 °C, the gas temperature was set to 230 °C, the gas flow was set to 10 L/min, the nebulizer was set to 3.1 bar, the voltage was set to 2500 V, and the polarity was negative for all compounds examined. The acquisition method was MRM with 8 product ions, a dwell time of 50 s, injection volume of 5 µL, and needle wash of 10 s.

Additional PFAS solution concentration analysis was performed courtesy of the University of Kentucky Mass Spectrometry Analytical Core. For targeted analysis of PFOA and PFBA aqueous solutions, samples were processed using a Thermo Scientific TSQ Altis Plus coupled with Vanquish Horizon HPLC.

## 4. Conclusions

The burgeoning impact of perfluorinated compounds requires scientists and researchers to examine and develop new remediation strategies that are effective, feasible, and environmentally friendly. Among the likely candidates for the capture and concentration of a variety of PFASs are amine-containing ion exchangers, although their application for large-scale water treatment is in its infancy. This work builds upon existing membrane frameworks and contaminant removal techniques to analyze the efficacy of anion-exchange membrane systems with regard to “forever chemicals” and their derivatives. A robust, scalable, water-based membrane synthesis was developed and employed to demonstrate the possible use of amine-containing ion-exchange resins within membrane pores. In situ polymerization allowed for pore functionalization as high as 0.85 and 3.4 mmol/g membrane for quaternary amines and primary amines, respectively. This technique is not limited to the use of polyvinylidene difluoride microfiltration membranes, bolstering its ability to be tailored for specific use cases outside of the focus of this work. The functionalized membranes were capable of over 50 LMH/bar at relatively low operating conditions. Furthermore, the synthesized membranes were of comparable and, in some instances, greater ion-exchange capacity, with quaternary and primary amines exhibiting 90 and 99% sorption of approximately 100 ppb PFOA, respectively. These qualities could be further enhanced through the optimization of charge density via synthesis alterations or by optimizing water and contaminant flux via operating conditions. Initial effluent concentrations were reduced to levels ranging from 10 ppb to below the limit of quantification of LC/MS/MS. This performance was maintained in the capture and desorption of PFOA and PFBA over three cycles. Understanding the ability to regenerate such membranes is vital to their competence and implementation in water treatment, addressing the issue of many remediation technologies that lead to more complex contamination needs. Overall, the research herein seeks to advance the state of technology available for the sequestration and eventual detoxification of per- and polyfluorinated alkyl substances that have become pervasive.

## Figures and Tables

**Table 1:** PFAS concentrations in environmental, academic, and industrial studies. Initial and residual concentrations correspond to levels before and after treatment, when applicable. Here, 1 ng/L = 0.001 ppb. Note: n/a means not applicable.

Location	Initial Levels (ng/L)	Residual Stream (ng/L)	Source
Tap water/ground water	2.3–8.4 *	n/a	[20]
	13 *	n/a	[21]
	54.7–248 **	n/a	[22,23]
Membrane filtration (RO or NF)	1000 *	10–50	[24]
	1,000,000 *	~100,000	[25]
Capture via molecular interactions (GAC, biochar, or IX)	4–18 *	1.56–7.02	[26]
	146 **	8.76	[27]
	430 *	n/a	[28]
	25.4 *	10.6 ± 2.4	[29]
Degradation (electrolysis/catalysis)	62 *	50.8	[30]
	30,000,000 **	23,100,000	[31]

Residual stream data estimated from reported removal efficiencies when applicable. RO: reverse osmosis; NF: nanofiltration; GAC: granular activated carbon; IX: ion exchange. * PFOA; ** ΣPFAS.

## Data Availability

Data are contained within the article or the Supplementary Materials, except for LC/MS/MS chromatograms, which will be made available by the authors upon request.

## Supplementary Materials

The following supporting information can be downloaded at: https://www.mdpi.com/article/10.3390/molecules29174229/s1, Figure S1: Membrane synthesis workflow; Figure S2: Water contact angle on pristine and functionalized membranes; Figure S3: Membrane permeance chart; Table S1: Membrane weight gain. Figure S4: FTIR analysis of membrane permeate.
