# Supplementary material for: Microfiltration Membrane Pore Functionalization with Primary and Quaternary Amines for PFAS Remediation: Capture, Regeneration, and Reuse
Source: Molecules. 2024 Sep 6;29(17):4229. doi: 10.3390/molecules29174229 (PMC11397369; doi:10.3390/molecules29174229)
Supplement: Supplementary file 1 [file molecules-29-04229-s001.zip › molecules-3161320-supplementary.pdf]

Microfiltration Membrane Pore Functionalization with Primary and Quaternary Amines for PFAS Remediation: Capture, Regeneration, and Reuse

Supplementary Information

Sam Thompson [1], Angela M. Gutierrez [2], Jennifer Bukowski [1] and Dibakar Bhattacharyya [1,*]

[1] Department of Chemical and Materials Engineering, University of Kentucky, Lexington, KY 40506, USA
[2] Sustainability and Analytical Equipment Facility, University of Kentucky, Lexington, KY 40506, USA
* Correspondence: db@uky.edu

Table of Contents

Figure S1: Membrane Synthesis Workflow

Figure S2: Water Contact Angle on Pristine and Functionalized Membranes

Figure S3: Pristine and Functionalized Membrane Pure Water Permeance

Figure S4: FTIR Analysis of Permeate and Residual Retentate

Table S1: Membrane Functionalization Details

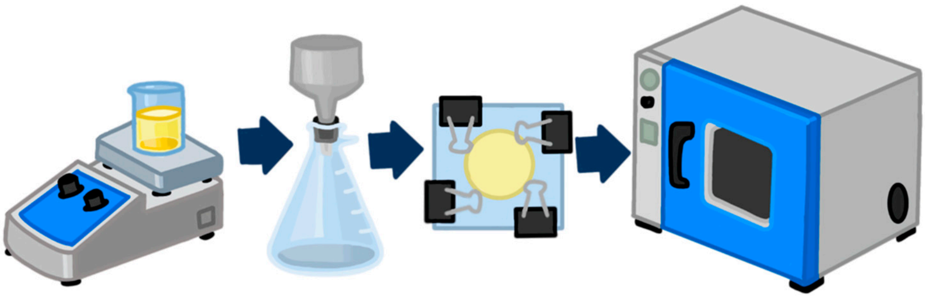

Figure S1. Workflow of Functionalized Membrane Synthesis. An aqueous solution containing monomer, crosslinker and initiator is mixed and convectively flowed through commercial microfiltration membranes via vacuum filtration. Excess solution is removed before clamping the membrane between insulating plates, whereupon the membrane is placed in a vacuum over at 85 °C for 3 hrs.

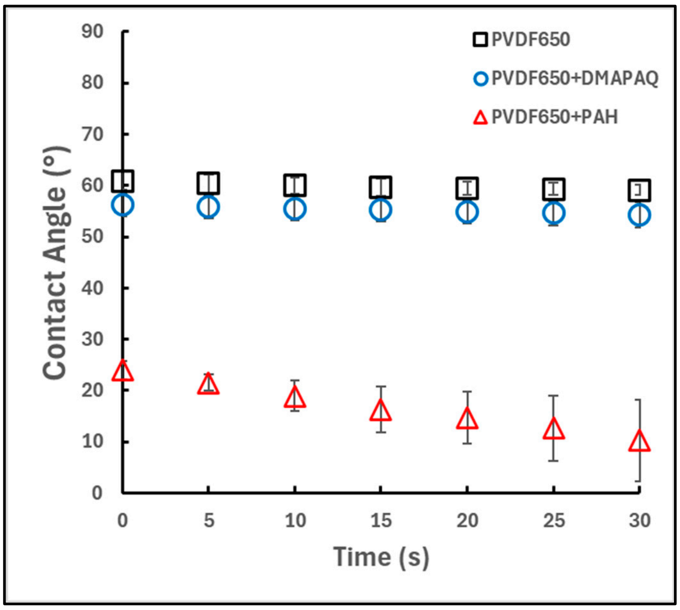

Figure S2. Water contact angle decay on pristine and functionalized PVDF membranes. Error bars represent +/- one standard deviation in series of contact angle measurements.

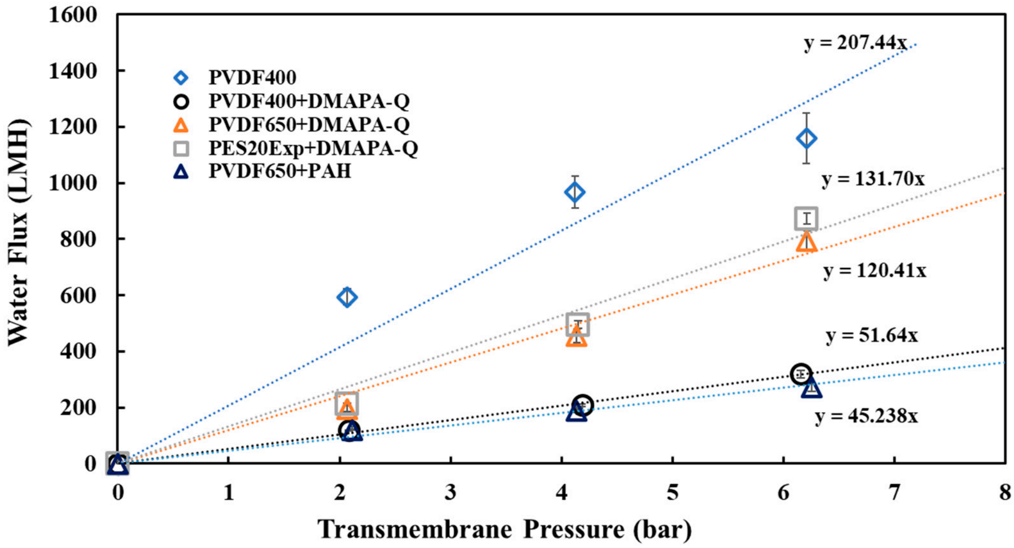

Figure S3. Pure water fluxes through pristine and functionalized membranes at various operational pressures. Regression lines corroborate to calculated permeance. Neutral pH and room temperature water used.

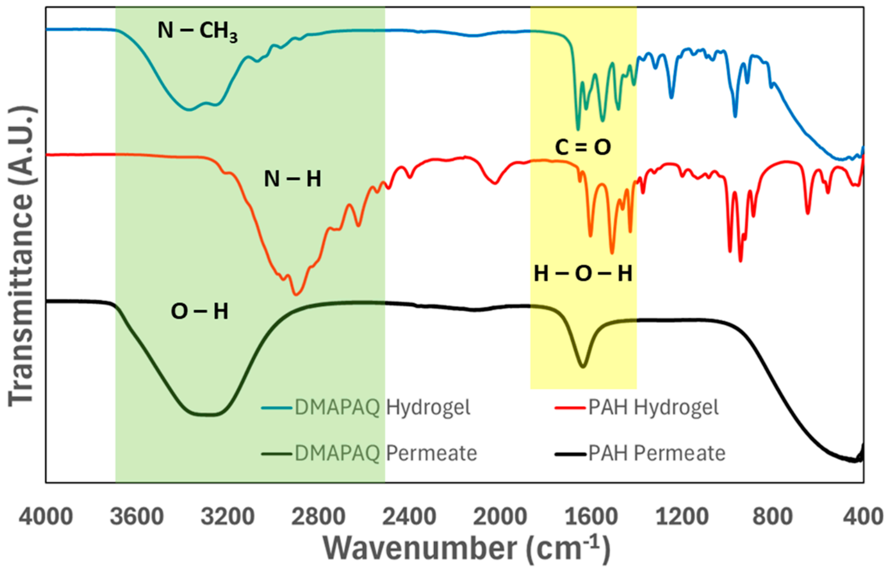

Figure S4. FTIR spectra of permeate and retentate after processing with functionalized membranes. Permeate spectra are overlapping for comparison.

Table S1. Membrane weight gain due to functionalization

Membrane	Polymer Synthesized	Pristine Weight (g)	Weight Gain (%)	N Groups in Membrane (mol N/g membrane)
PVDF400	DMAPAQ	0.604	3.974	0.000246
		0.612	2.288	0.000142
		0.615	4.228	0.000262
		0.618	5.178	0.000321
PVDF650	DMAPAQ	0.611	4.092	0.000254
		0.624	13.622	0.000845
		0.611	4.092	0.000254
		0.624	13.622	0.000845
		0.563	4.085	0.000253
		0.598	3.512	0.000218
		0.584	7.534	0.000467
		0.57	5.088	0.000316
		0.587	6.303	0.000391
		0.59	5.085	0.000315
		0.593	5.059	0.000314
		0.598	4.348	0.000270
	PAH	0.593	27.825	0.00297
		0.585	21.538	0.00230
		0.6	11.167	0.00119
		0.608	31.579	0.00338
		0.589	23.939	0.00256
		0.599	9.182	0.000981
		0.592	11.486	0.00123
		0.62	20.000	0.00214
		0.597	13.065	0.00140
		0.604	9.106	0.000973
PES Express Plus	DMAPAQ	0.317	39.117	0.00243
